# The important effect of 5-HTTLPR polymorphism on the risk of depression in patients with coronary heart disease: a meta-analysis

**DOI:** 10.1186/s12872-020-01424-1

**Published:** 2020-03-18

**Authors:** Li-Jun Zhang, Xian-Tao Zeng, Ming-Juan Zhao, Dong-Fang He, Jian-Yang Liu, Mei-Yan Liu

**Affiliations:** 1 Department of Cardiology, Beijing Anzhen Hospital Affiliated to Capital Medical University, No. 2 Anzhen Road, Chaoyang District, Beijing, 100029 China; 2 Center for Evidence-Based and Translational Medicine, Zhongnan Hospital of Wuhan University, Wuhan, 430071 Hubei China; 3 Center for Evidence-Based and Translational Medicine, Wuhan University, Wuhan, 430071 Hubei China; 4 Department of Cardiology, The First Affiliated Hospital of Henan University, Kaifeng, 475001 Henan China

**Keywords:** 5-HTTLPR polymorphism, Depression, Coronary heart disease, Meta-analysis

## Abstract

**Background:**

Depression has been recognized as an independent risk factor of coronary heart disease (CHD). Moreover, there is interrelationship of both depression and CHD. However, the potential pathophysiological mechanisms remain unknown. It might be influenced by genetic and environmental factors. According to recent researches, there is potential association between serotonin transporter gene-linked polymorphic region (5-HTTLPR) polymorphism and risk of depression in CHD patients, but the results are still inconclusive. Therefore, we performed this meta-analysis based on unadjusted and adjusted data to ascertain a more precise conclusion.

**Methods:**

We searched relevant articles through PubMed, Embase, Web of Science, Chinese BioMedical Literature (CBM) and Chinese National Knowledge Infrastructure (CNKI) databases up to August 26, 2019. Study selection and data extraction were accomplished by two authors independently. The strength of the correlation was assessed via odds ratios (ORs) with their 95% confidence intervals (95%CIs).

**Results:**

This meta-analysis enrolled six observational studies. Based on unadjusted data, there was significant relationship between 5-HTTLPR polymorphism and depression risk in CHD patients under all genetic models (S vs. L: OR = 1.31, 95%CI = 1.07–1.60; SS vs. LL: OR = 1.73, 95%CI = 1.12–2.67; LS vs. LL: OR = 1.47, 95%CI = 1.13–1.92; LS + SS vs. LL: OR = 1.62, 95%CI = 1.25–2.09; SS vs. LL + LS: OR = 1.33, 95%CI = 1.02–1.74). The results of adjusted data further strengthened this relationship (SS vs. LL: OR = 1.89, 95%CI = 1.28–2.80; LS vs. LL: OR = 1.69, 95%CI = 1.14–2.51; LS + SS vs. LL: OR = 1.80, 95%CI = 1.25–2.59). Subgroup analyses based on ethnicity and major depressive disorder revealed similar results to that of the overall analysis. No evidence of publication bias was observed.

**Conclusions:**

Our results suggest that 5-HTTLPR polymorphism may have an important effect on the risk of depression among patients with CHD, and carriers of the S allele of 5-HTTLPR are more vulnerable to depression.

## Background

Depression has taken a pivotal place in contributing to coronary heart disease (CHD), what’s worse, the comorbidity accounts for an increasing trend of mortality [1–4]. There is no reason to ignore its threat to human health. However, the comorbidity is regarded as interdisciplinary science that associates psychology, psychiatry, neurology and cardiology, which increases complexity and difficulty of revealing the underlying mechanisms. According to the related researches, the potential mechanisms involve the dysfunction of hypothalamus-pituitary-adrenal aixs, dysregulation of sympathetic and parasympathetic nerves system, dysmodulation of special neurotransmitters or hormones likely serotonin (5-HT), adrenalin [5, 6].

Notably 5-HT, it could be considered as a good communicator between central nervous system (CNS) and peripheral system, due to its signal transporting role in CNS transduction when releasing by presynapic vesicles, and its vasoconstricting and vasodilating role in periphery when releasing by platelets [7]. Then we must mention another vital factor - 5-HT transporter (5-HTT) which is responsible for the reuptake of 5-HT and regulates the balance of intracellular and extracellular 5-HT. 5-HTT is a kind of transmembrane protein belonged to solute carrier family, and encoded by SLC6A4 gene localised in chromosome 17q11.1-q12 [8]. Moreover, the 5-HTT gene-linked polymorphic region (5-HTTLPR) polymorphism has achieved particular attention, owing to its direct role in influencing the transcriptional activity of SLC6A4 gene. 5-HTTLPR polymorphism involves allele deletion (the short allele, S allele) and allele insertion (the long allele, L allele). S allele is demonstrated to have lower transcriptional activity than L allele, resulting in the reduction of 5-HTT in the cell membrane [9, 10]. Consequently, the reduction of 5-HTT causes the imbalance of 5-HT concentration and function, which is believed to be one of the important causation of depression and accelerates the progress of CHD [9, 10].

Several individual studies have conducted on the association of 5-HTTLPR and depression in CHD patients. For instance, Nakatani et al. [11] indicated that S allele was related with depressive symptoms in patients with acute myocardial infarction (AMI), otherwise, they found S allele was correlated with cardiac events in the follow-up study. While different conclusions were elucidated in both Cao [12] and Bozzin’ s [13] researches. In order to have a comprehensive understanding of the relation between 5-HTTLPR and depression in CHD patients, we performed this meta-analysis and subgroup analysis according to race and major depression (MD).

## Methods

We performed this meta-analysis according to the requirements of PRISMA (Preferred Reporting Items for Systematic Reviews and Meta-Analyses) [14]. There was no need for ethical approval. We had registered in PROSPERO for this meta-analysis, and the number was CRD42017074196.

### Eligibility criteria

Studies would be selected according to the following criteria: (1) Patients with established CHD, combined with depression in case group, contrarily in control group; (2) 5-HTTLPR had been detected; (3) Cohort, case-control or cross-sectional study; (4) English or Chinese language; (5) Data were eligible to extract and full-texts could be found. If two or more studies involved the same population, we would select the one with most recent or complete study.

### Search strategy

Articles published before August 26, 2019, were collected from PubMed, Embase, Web of Science, Chinese BioMedical Literature (CBM) and Chinese National Knowledge Infrastructure (CNKI) databases. Search terms: (coronary disease OR myocardial infarction OR coronary heart disease OR CHD OR acute coronary syndrome OR coronary artery disease) AND (depression OR depressive disorder OR depressive symptoms OR depressive syndrome) AND (Serotonin transporter OR 5-hydroxytryptamine transporter OR 5-HT transporter OR 5-HTT OR SLC6A4 OR 5-HTT gene–linked polymorphic region OR 5-HTTLPR) AND (polymorphism OR SNP OR Variant). In addition, hand search was conducted for relevant studies from reference lists.

### Data extraction

Two authors were responsible for data extraction of the enrolled articles. They independently extracted necessary information in accordance with a standard table including: surname of first author, publication year, country and ethnicity, study design, diagnostic criteria for depression, genotyping method, sample size, genotype distributions in depressed and non-depressed CHD patients, the value of adjusted OR and its 95%CI and adjusted variables.

### Statistical analysis

The correlation strength was assessed by odds ratios and the corresponding 95% confidence intervals (95% CIs). For crude data, we calculated ORs and their 95%CIs according to the allele comparison (S vs. L), homozygote comparison (SS vs. LL), heterozygote comparison (LS vs. LL), dominant model (LS + SS vs. LL), and recessive model (SS vs. LL + LS). For adjusted data, the relevant ORs and their 95%CIs were able to be found in the article. We measured heterogeneity assumption by Cochrane *Q* test and I^2^ statistic [15]. Random-effects model (*P* < 0.10 or I^2^ > 50%) or fixed-effect model (*P* > 0.10 and I^2^ < 50%) would be used according to the significant heterogeneity.

Subgroup analyses were performed based on ethnicity and major depressive disorder. All above analyses were performed using the Review Manager 5.3 software. In addition, Egger’s tests [16] and funnel plots were used for assessing potential publication bias by STATA 12.0 software.

## Results

### Characteristics of included studies

**Fig. 1:**
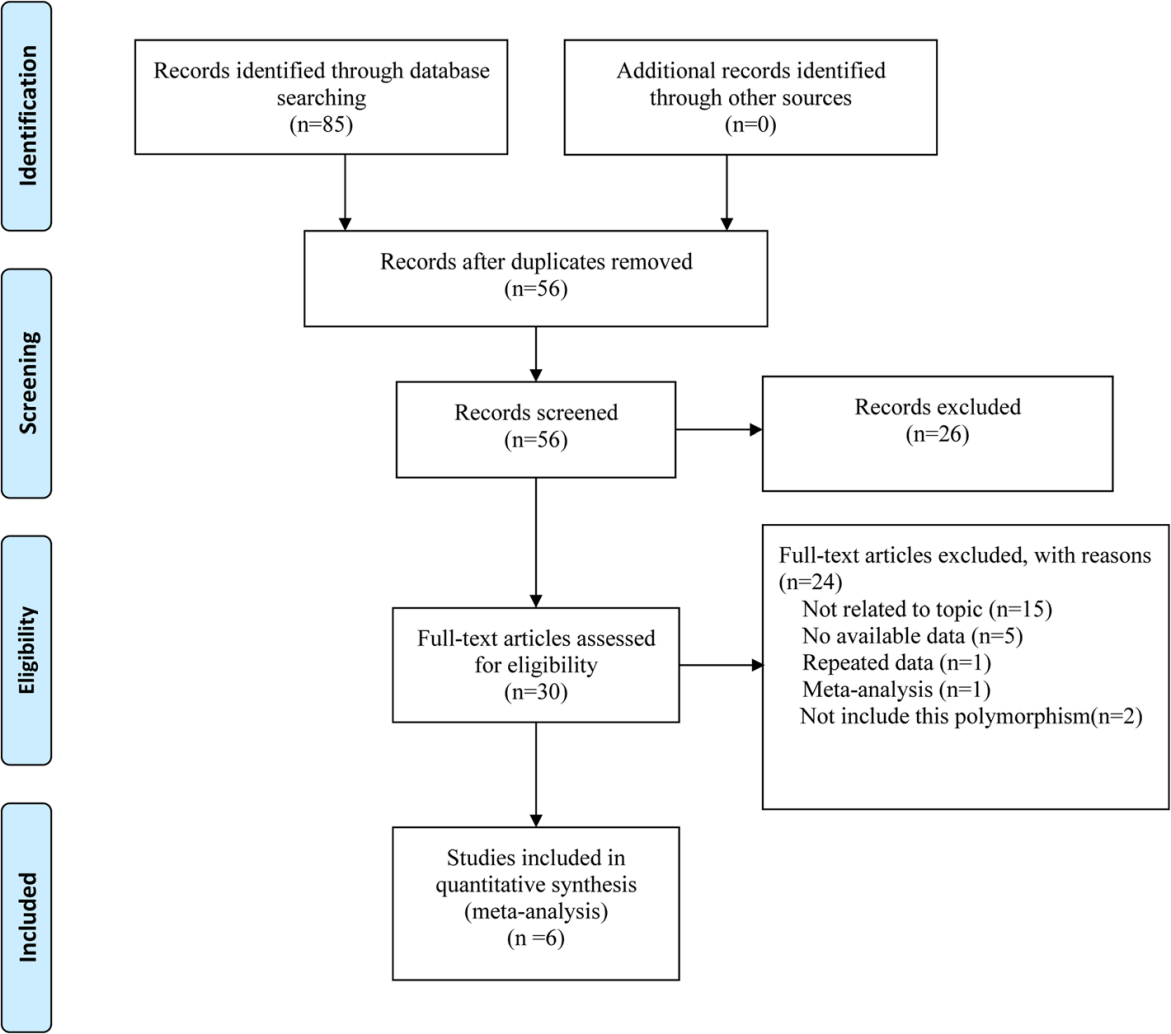
The flow diagram of study selection

A total of 85 records was yielded and finally 6 observational studies [11, 12, 17–20] were selected in this meta-analysis, involving 1780 CHD comorbid depression (CHD-D) patients and 2712 CHD without depression (CHD-nD) patients. A flow diagram of the process of study selection was presented in Fig. 1.

Of them, there were two case-control studies conducted in China [12, 17]; two prospective observational studies conducted in Japan [11] and Korea [20], respectively. Another two studied patients enrolled in the same prospective observational study in America [18, 19], but one of the studies focused on major depressive disorder [19]. Regarding the prospective studies, we only extracted the baseline genotype data for analysis. Indeed, they can be deemed as case-control studies in this meta-analysis. The study by Kim et al. [20] investigated both any and major depressive disorder. Finally, five studies [11, 12, 17, 18, 20] were involved in overall analysis and two studies [19, 20] involved in investigating major depressive disorder. Furthermore, five studies [11, 12, 17, 18, 20] provided the distribution of the polymorphism in depressed and non-depressed CHD patients as well as adjusted ORs and their 95%CIs. No deviation from the Hardy-Weinberg equilibrium was observed for any genotype. Blood samples were collected and polymerase chain reaction (PCR)-based genotyping was used to detect the polymorphism in all eligible studies. Detailed information of characteristics of included studies is summarized in Tables 1 and 2.

**Table 1:** Characteristics and unadjusted data of included studies

Reference	Country (Ethnicity)	Diagnosticcriteria for depression	Severity of depression	Sample size		CHD-D			CHD-nD			Genotype method	HWE
				CHD-D	CHD-nD	S/S	L/S	L/L	S/S	L/S	L/L		
Nakatani 2005 [11]	Japan (Asian)	SDS	Depression	861	942	552	281	28	592	298	52	PCR	Yes
Cao 2007 [12]	China (Asian)	CCMD-3; SDS; HAMD	Depression	153	171	95	46	12	96	67	8	PCR	Yes
Xia 2006 [17]	China (Asian)	CCMD-3; SDS	Depression	70	70	35	25	10	16	32	22	PCR	Yes
Otte 2007 [18]	America (White)	Interviews applying for DSM-IV criteria.	Depression	126	431	24	73	29	72	214	145	PCR	Yes
Kangelaris 2010 [19]	America (Mixed)	Interviews applying for DSM-IV criteria.	MDD	192	678	35	102	55	137	314	227	PCR	Yes
Kim 2015 [20]	Korea (Asian)	Interviews applying for DSM-IV criteria.	Depression	378	591	239	121	18	329	219	43	PCR	Yes
			MDD	177	591	119	53	5	329	219	43	PCR	

*CHD-D* coronary heart disease comorbid depression, *CHD-nD* coronary heart disease without depression; Mixed,White, African American, Asian, Latino, Other; *HWE* Hardy-Weinberg equilibrium, *PCR* Polymerase Chain Reaction

*SDS* The Zung Self-rating Depression Scale, *HAMD* Hamilton Depression Scale, *DSM-IV* Diagnostic and Statistical Manual of Mental Disorders, 4th Edition

**Table 2:** Adjustment and adjusted data of included studies

Reference	Ethnicity	Gentic models	OR(95%CI)	Adjustment
Nakatani 2005 [11]	Asian	SS vs LL	1.97 (1.16–3.35)	age, sex, diabetes mellitus, hypertension, hyperlipidemia, smoking, a history of MI, a Killip class of >II, anterior infarction, reperfusion therapy, and treatment with antiplatelet agents, angiotensin-converting enzyme inhibitors, or beta-blockers.
		LS vs LL	2.02 (1.17–3.51)	
		LS + SS vs LL	2.19 (1.21–3.98)	
Otte 2007 [18]	White	LS + SS vs LL	1.6 (1.0–2.5)	age, sex
[17]	Asian	SS vs LL + LS	2.048 (1.132–3.704)	hypertension, smoking, diabetes, BMI, TC, TG, LDL-C, HDL-C,
Cao 2007 [12]	Asian	S vs L	2.34 (1.38–3.98)	age, sex, smoking, diabetes,, education, hypertension, TC, TG
Kim 2015 [20]	Asian	SS vs LL	1.80 (1.03–3.30)	sex, education, living alone, housing, current employment, hypertension, diabetes, and current smoking.
		LS vs LL	1.39 (0.79–2.49)	

### Overall analyses

**Fig. 2:**
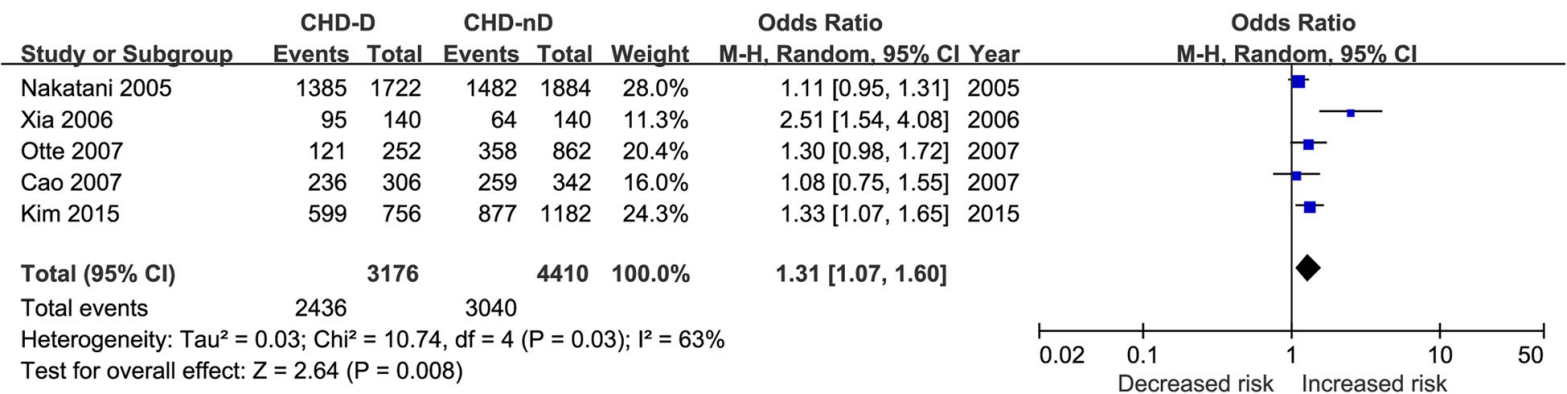
Forest plot of overall analysis in allele comparison. CHD-D, coronary heart disease comorbid depression; CHD-nD, coronary heart disease without depression; CI, confidence intervals

**Fig. 3:**
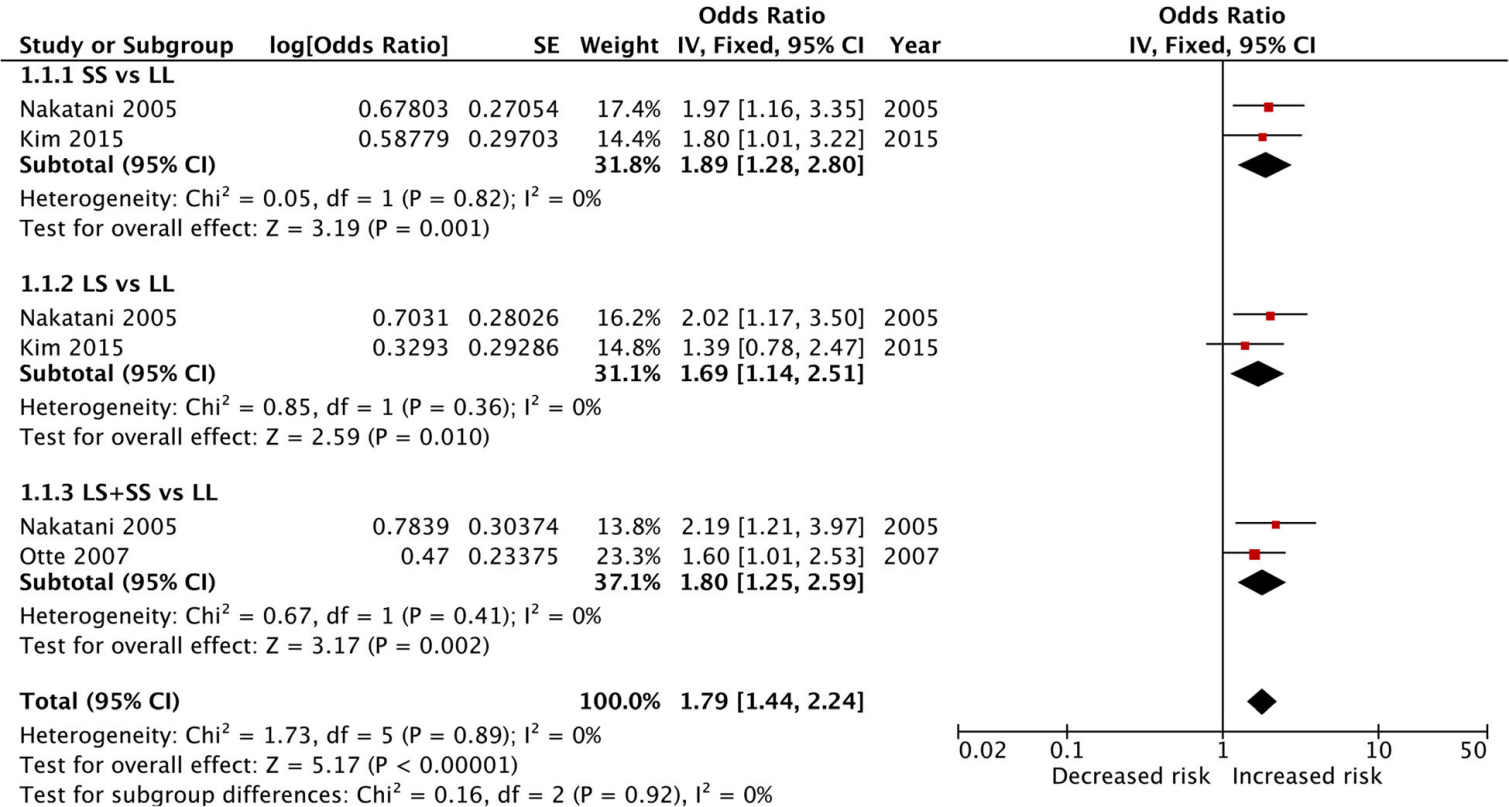
Forest plot of adjusted data in all five genetic models. CI, confidence intervals

The pooled results from crude data indicated there was remarkable association between 5-HTTLPR polymorphism and depression risk in CHD patients under all genetic models [S vs. L: OR = 1.31, 95%CI = 1.07–1.60 (Fig. 2); SS vs. LL: OR = 1.73, 95%CI = 1.12–2.67; LS vs. LL: OR = 1.47, 95%CI = 1.13–1.92; LS + SS vs. LL: OR = 1.62, 95%CI = 1.25–2.09; SS vs. LL + LS: OR = 1.33, 95%CI = 1.02–1.74] (Table 3). The results of adjusted data strengthened the association between 5-HTTLPR polymorphism and depression risk in CHD patients (SS vs. LL: OR = 1.89, 95%CI = 1.28–2.80; LS vs. LL: OR = 1.69, 95%CI = 1.14–2.51; LS + SS vs. LL: OR = 1.80, 95%CI = 1.25–2.59) (Fig. 3).

**Table 3:** Results of overall and subgroup analyses

Overall and subgroup analysis	Studies	Test of association		Test of association	
		OR(95%CI)	***P***-value	I^**2**^	P***h***
**S vs. L**					
Total	5	1.31 (1.07–1.60)	0.008	63%	0.03
Asian	4	1.33 (1.03–1.72)	0.03	72%	0.01
White	1	1.30 (0.98–1.72)	0.07	NA	NA
Major depressive disorder	2	1.29 (0.86–1.94)	0.22	79%	0.03
**SS vs. LL**					
Total	5	1.73 (1.12–2.67)	0.01	53%	0.08
Asian	4	1.75 (0.97–3.13)	0.06	65%	0.04
White	1	1.67 (0.91–3.07)	0.10	NA	NA
Major depressive disorder	2	1.67 (0.58–4.83)	0.34	76%	0.04
**LS vs. LL**					
Total	5	1.47 (1.13–1.92)	0.005	40%	0.15
Asian	4	1.27 (0.77–2.10)	0.35	51%	0.11
White	1	1.71 (1.06–2.75)	0.03	NA	NA
Major depressive disorder	2	1.43 (1.01, 2.02)	0.04	0%	0.41
**LS + SS vs. LL**					
Total	5	1.62 (1.25–2.09)	0.0002	38%	0.17
Asian	4	1.52 (0.93–2.47)	0.09	53%	0.09
White	1	1.70 (1.07–2.69)	0.02	NA	NA
Major depressive disorder	2	1.62 (0.79–3.30)	0.19	56%	0.13
**SS vs. LL + LS**					
Total	5	1.33 (1.02–1.74)	0.03	62%	0.03
Asian	4	1.39 (1.01–1.91)	0.05	71%	0.02
White	1	1.17 (0.70–1.96)	0.54	NA	NA
Major depressive disorder	2	1.21 (0.66–2.22)	0.54	80%	0.03

*OR* odds ratio, *CI* confidence intervals, *NA* not applicable

### Subgroup analyses

The subgroup analyses results were similar to that of the overall analysis. Significant associations were observed in Asians [S vs. L: OR = 1.33, 95%CI = 1.03–1.72; SS vs. LL + LS: OR = 1.39, 95%CI = 1.01–1.91] and Whites [LS vs. LL: OR = 1.71, 95%CI = 1.06–2.75; LS + SS vs. LL: OR = 1.70, 95%CI = 1.07–2.69]. Statistical association between 5-HTTLPR polymorphism and major depressive disorder in CHD patients was also observed [LS vs. LL: OR = 1.43, 95%CI = 1.01–2.02]. All the results are presented in Table 3.

### Publication bias

**Fig. 4:**
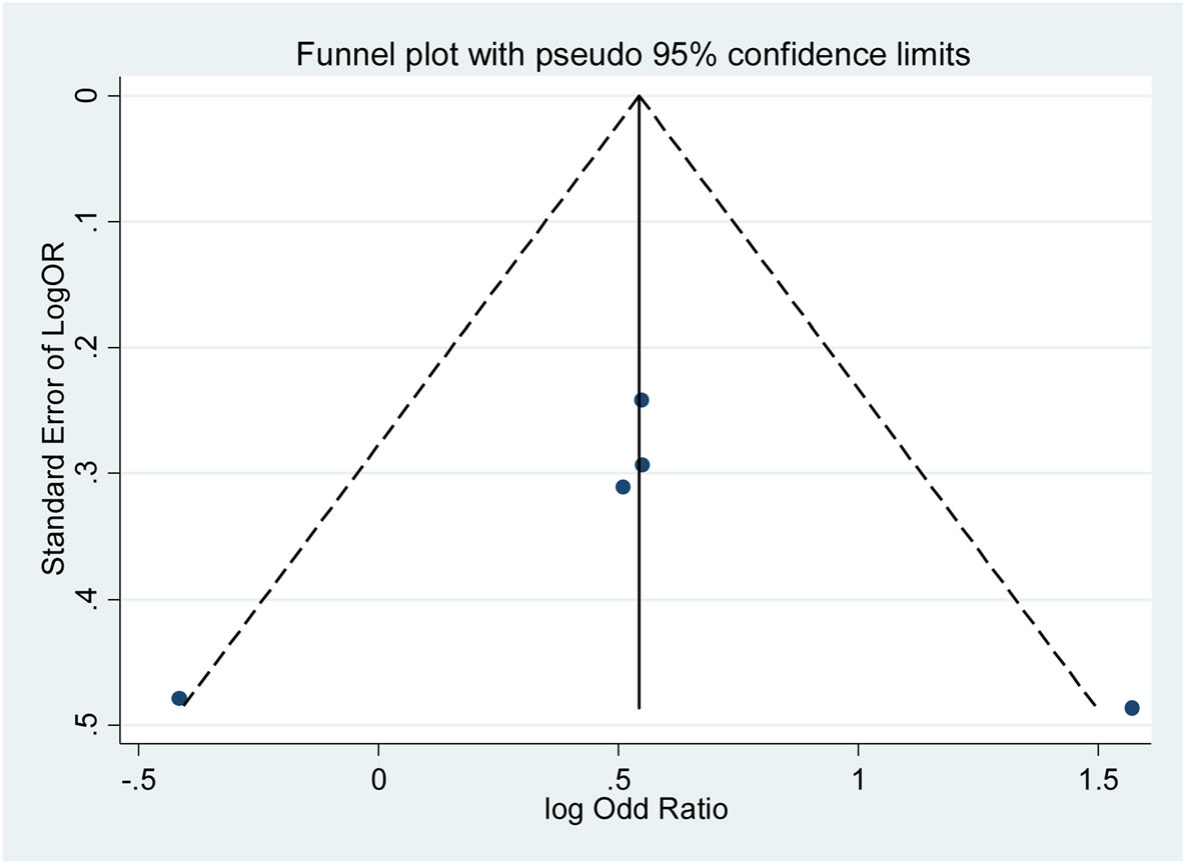
Funnel plot of overall analysis in homozygote comparison. OR, odds ratio

In this meta-analysis, no evidence of publication bias was detected under any genetic models assessed by Egger’s test (S vs. L: *p* = 0.196; SS vs. LL: *p* = 0.962; LS vs. LL: *p* = 0.195; LS + SS vs. LL: *p* = 0.543; SS vs. LL + LS: *p* = 0.151) and funnel plots (Fig. 4).

## Discussion

In accordance with Nakatani [11], Xia [17], Otte [18], and Kim [20], this meta-analysis elucidates that S allele (including SS and SL) is related with depression in CHD patients, especially major depression. Otherwise, subgroup analysis explains that S allele associates with both Asian and White. The implications are worth special considerations.

As far as we are concerned, depression and CHD are not isolated form each other, but interrelated with each other essentially. In the light of the current researches, we hypothesis the potential mechanisms under 5-HTTLPR, depression and CHD as follows:

Firstly, CHD patients with S allele present expression reduction of 5-HTT in the cell membrane inducing decreased reuptake of 5-HT. Moreover, the total concentration of 5-HT in synaptic cleft reduces in virtue of organic cation transporter (OCT) [21], which has been proved to reuptake 5-HT in compensation of 5-HTT reduction. Consequently, depressive symptoms develop due to the deficiency of 5-HT, which could be treated with selective serotonin reuptake inhibitor (SSRI) by targeting at 5-HTT. In this meta-analysis, we indicate the association of S allele and depression, notably major depression. The major strength of this study is that we consider adjusted data and severity of depression. According to Diagnostic and Statistical Manual of Mental Disorders Fifth Edition (DSM-5), depressive disorders involve major depressive disorder, persistent depressive disorder, depressive disorder due to another medical condition et al. [22]. Two studies from Kangelaris [19] and kim [20] respectively, focus on major depression, drawing the identical conclusion that S allele increases the risk of major depression in CHD patients.

Secondly, 5-HT, as a neurotransmitter in CNS, transduces biological signal via activating 5-HT receptors located in postsynaptic membrane. Notably 5-HT_2A_ receptor (5-HT_2A_R) is a dominant receptor of serotonin receptor family. Our former researches have demonstrated the relationship between 5-HT_2A_R and myocardial infarction combined with depression [23]. Moreover, several studies reported the important role of 5-HT_2A_R gene polymorphisms (− 1438 A/G and 102 T/C) in psychiatric disorders [24]. While a meta-analysis didn’t find significant association between − 1438 A/G polymorphism of 5-HT_2A_R and major depressive disorders [25]. Therefore, more original researches should be done to further explore the relationship.

Thirdly, it is widely acknowledged that ethnicity is associated with genetic polymorphism [26]. This meta-analysis involves Asians (from China [12, 17], Japan [11], and Korea [20]) and Whites (from America [18, 19]). To explore the relationship between ethnicity and 5-HTTLPR polymorphism, we performed the subgroup analyses based on ethnicity. The results showed 5-HTTLPR polymorphism and depression risk in CHD patients of both Asians and Whites. (Table 3).

In addition, we have to admit the limitations in this meta-analysis. Firstly, only six original researches are eligible for this meta-analysis, making the results not roust enough. Hence, we are expecting for more original studies conducted on this topic; Secondly, it would be better to focus on sex specificity for the differences of 5-HT synthesis rate between female and male. However, the current data collected from the studies don’t support sex subgroup analysis; Thirdly, for sake of exploring the possible mechanisms, the relevant factors involving 5-HT, 5-HTT should be detected. While no studies had performed it.

## Conclusion

In conclusion, CHD patients with S allele are more vulnerable to depression, even major depression. However, we still need more evidence to prove the its specifity and sensitivity before it could be adapted to clinical diagnose. We believe that, in the near future, we could predict depression in CHD patients before they present any clinical symptoms just by extracting a few blood for 5-HTTLPR detection.

## Data Availability

The data can be obtained from the corresponding author under reasonable request.

## Abbreviations

CHD: Coronary heart disease
5-HTTLPR: Serotonin transporter gene-linked polymorphic region
CNS: Central nervous system
CBM: Chinese BioMedical literature
CNKI: Chinese national knowledge infrastructure
ORs: Odds ratios
95%CIs: 95% confidence intervals
5-HT: Serotonin
5-HTT: Serotonin transporter
PCR: Polymerase chain reaction
HT_2A_R: Serotonin receptor 2A
DSM-5: Diagnostic and statistical manual of mental disorders fifth edition
CDIS-IV: Computerized diagnostic interview schedule
MINI: MINI-international neuropsychiatric interview
SDS: Zung self-rating depression scale
